# Prescribing patterns and economic costs of proton pump inhibitors in Colombia

**Published:** 2013-03-30

**Authors:** Jorge Machado-Alba, Alejandra Fernández, Juan Daniel Castrillón, Carlos Felipe Campo, Luis Felipe Echeverri, Andrés Gaviria, Manuel José Londoño, Sergio Andrés Ochoa, Joaquín Octavio Ruíz

**Affiliations:** a Grupo Investigación Farmacoepidemiología y Fármacovigilancia. Universidad Tecnológica de Pereira-Audifarma S.A, E-mail: alita1019@hotmail.com; b Universidad Autonoma de Barcelona. E-mail: machado@utp.edu.co

**Keywords:** Health care costs, drug costs, pharmaceutical services, omeprazole, esomeprazole, lanzoprazole

## Abstract

**Objective:**

To determine the prescribing patterns for proton pump inhibitors and to estimate the economic cost of their use in a group of patients affiliated with the Colombian Health System.

**Methods:**

This is a descriptive observational study. Data for analysis consisted of prescriptions dispensed between October 1st, 2010 and October 31st, 2010 and were collected from a systematic database of 4.2 million members. Socio-demographic variables were considered along with the defined daily dose,comedication, convenience of the indication for proton pump inhibitor use and costs.

**Results:**

In this study, 113,560 prescriptions were dispensed in 89 cities, mostly to women (57.6%) with a mean age of 54.4 ± 18.7 years; the drugs were omeprazole (n= 111.294; 97.81%),esomeprazole (n= 1.378; 1.2%), lansoprazole (n= 524; 0.4%), pantoprazole and rabeprazole. The indication for 87.349 of the formulas (76.9%) was justified and statistically associated with the use of NSAIDs, antithrombotics, corticosteroids, anti-ulcer, antibiotics and prokinetics. No justification was found for 26.211 (23.1%) of the prescriptions, which were associated with antidiabetics, antihypertensives, hypolipidemics and others (*p* <0.001).The annual justified cost was estimated to be US$ 1,654,701 and the unjustified cost was estimated to be U.S. $2,202,590, as calculated using the minimum reference prices.

**Discussion:**

Each month, the Colombian health system is overloaded by unjustified costs that include payments for non-approved indications of proton pump inhibitors and for drugs outside the list of essential medications. This issue is contributing to rising costs of healthcare in Colombia.

## Abstract (es)

**Objetivos:**

Determinar los patrones de prescripción de inhibidores de la bomba de protones y estimar el costo económico que genera su utilización en pacientes afiliados al Sistema General de Seguridad Social en Salud de Colombia.

**Metodología:**

Estudio descriptivo observacional. Se analizarondatos de formulas dispensadas entre 1 y 31 de octubre de 2010 de una base de datos sistematizada de 4.2 millones de afiliados.Se consideraron variables socio-demograficas, dosis diaria definida,comedicación recibida, conveniencia o no del tipo de indicación de IBP y costos.

**Resultados:**

Se dispensaron 113 560 formulas en 89 municipios,principalmente a mujeres (57.6%); promedio de edad 54.4±18.7 años; los medicamentos fueron omeprazol (n= 111294, 97.8%),esomeprazol (n= 1378, 1.2%), lanzoprazol (n= 524, 0.4%), pantoprazol y rabeprazol a dosis diarias definidas adecuadas. Se halló justificación en la indicación de 87349 fórmulas (76.9%) asociadas estadísticamente con uso de AINEs, antitrombóticos, corticoides, antiulcerosos, antibióticos, procinéticos, y sin justificación 26211 (23.1%) asociadas a antidiabéticos, antihipertensivos e hipolipemiantes y otros (*p* <0.001). El costo anual justificado fue de US$1.654.701 y no justificado de U.S.$2,202,590 empleando mínimos precios de referencia.

**Discusión:**

Mensualmente se está cargando al sistema de salud colombiano con costos no justificados del uso de inhibidores de bomba de protones en indicaciones no aprobadas y con medicamentos por fuera del listado de esenciales que contribuyen a encarecer la atención sanitaria.

## Introduction

Proton pump inhibitors (PPIs) were identified in 1979 and approved for the management of acid-peptic disease; they were subsequently introduced to the market in 1989^1^ . Several recent studies comparing omeprazole and ranitidine demonstrate the greater effectiveness of omeprazole in the treatment of peptic ulcer disease^2^, upper gastrointestinal bleeding^1^ ^,^ ^2^ and gastroesophageal reflux, leading to an increase in the use of PPIs^3^. In some cases, however, off-label uses increase PPI sales^3^ ^-^ ^5^and increase the overall costs for healthcare worldwide^6^ ^,^ ^7^ ^.^

Several conditions justify the use of PPIs, including different forms of peptic ulcer disease (Helicobater pylori associated or not), functional dyspepsia, gastroesophageal reflux, gastrointestinal bleeding prevention in conditions of severe stress and prophylaxis for peptic ulcer disease induced by non-steroidal anti inflammatory drugs (NSAIDs) and corticosteroids. However, the use of PPIs has begun to extend to pathologies for which they were not designed and for which there is insufficient scientific evidence justify their use^6^ ^-^ ^8^.

The uncontrolled use of PPIs is associated with atrophic gastritis, interstitial nephritis, induction of ulcer symptoms, thrombocytopenia, osteoporosis and endocrine disorders such as gynecomastia and impotence^9^ ^-^ ^12^. The probability of adverse reactions to PPIs also increases with polymedication and is higher in patients with chronic diseases^13^ ^,^ ^14^. This is due in part to the metabolism of PPIs through cytochrome P450, which leads to various drugs interactions by extending their half-life and thereby causes harmful systemic effects^14^.

Countries such as Argentina, Ireland, Spain and Greece have reported significant additional healthcare costs incurred by the inappropriate prescription of PPIs. Indeed, it has been found that between 70% and 80% of PPIs prescriptions are for off-label uses ^3^ ^,^ ^4^ ^,^ ^6^ ^,^ ^8^. To date, this type of study had not been done in Colombia, but is necessary in order to determine the pattern of PPI prescriptions and their costs, just as previous research has done for antihypertensives, antidiabetics, lipid lowering drugs, anti-tumor necrosis factor, antibiotics and antiretrovirals. Studies on all of these medications have revealed issues with dosing, indications, safety and cost effectiveness within the Colombian Health System (SGSSS)^15^ ^,^ ^16^.

In order to determine the prescribing patterns of PPIs and to calculate the costs generated by their use, this research was conducted in an outpatient population of the SGSSS that were being treated with these drugs. The implementation of information systems in investigating prescribing practices has been an essential tool for achieving a greater therapeutic quality of the drug prescriptions, contributing in improving the rationale use of drugs according to their approved indications.

## Materials and Methods

This is a descriptive study that provides an economic analysis of PPI prescription patterns in a sample of 4.2 million people that represent 20.4% of the patients affiliated with SGSSS and 8.2 % of the total population of Colombia.

These patients were affiliated with 16 different health insurance companies (EPS) and 42 different health service providers (IPS) across 89 cities of between 20,000 and 7.5 million inhabitants. The cities of Barranquilla, Bogota, Bucaramanga, Cali, Cartagena, Ibague, Manizales, Medellin and Pereira accounted for 80% of the patients. Patients of all ages and of both sexes who received a prescription for a PPI between October 1, 2010 and October 31, 2010 were included in this research sample.

The Department of Pharmacoepidemiology of the company that dispenses PPIs helped design a database allowing for the collection of certain descriptive variables about the patients using these medications, as described below:

Demographic variables recorded included age, gender, insurance company (EPS) provider, and city. Information was also collected on the specific PPI drugs used along with their respective doses; these drugs were esomeprazole, lansoprazole, omeprazole, pantoprazole and rabeprazole.

Information on comedications was also collected. Pertinent comedications included: a) Antihypertensives (ACE inhibitors, diuretics, beta blockers, calcium antagonists, prazosin), b) antiplatelet agents (acetylsalicylic acid, clopidogrel), c) analgesics (acetaminophen, metamizole), d) diabetes medications (insulin, metformin, sulfonilureas), e) NSAIDs (ibuprofen and others), f) antiemetics (metoclopramide), g) antibiotics for the treatment of H. pylori infection (amoxicillin, clarithromycin, azithromycin, metronidazole, tetracyclines, levofloxacin), h) corticosteroids (prednisolone, and others), i) lipid-lowering drugs (statins, fibrates, ezetimibe, cholestyramine), j) other anti-ulcer medications (sucralfate, bismuth, antacids, H2 blockers), k) Disease modifying anti rheumatic drugs (DMARDs) (methotrexate, and others), l) nitrates, m) inotropic agents (digoxin, metildigoxin), n) bisphosphonates, and o) antithyroid /thyroid hormone medications (methimazole, propylthiouracil, levotiroxin)^15^ ^,^ ^16^.

Among the PPI-treated patient pool comedication was used as surrogate indicator of chronic disease for the following classes of treatment and associated conditions: antihypertensive / high blood pressure, antiplatelet /ischemic heart disease, analgesics / pain management, antidiabetic drugs / diabetes mellitus, NSAID / inflammatory joint disease, antiemetics / reflux or dyspepsia, antibiotics / peptic ulcer disease by *H. pylori*, corticosteroid / inflammatory or autoimmune disease, lipid lowering drug / dyslipemia, PPIs or H2 blocker / peptic ulcer disease, DMARDs/ rheumatoid arthritis, nitrate / ischemic heart disease, inotropic agent/ heart failure or atrial fibrillation, bisphosphonates / osteoporosis, anti thyroid or thyroid hormone/ hyper or hypothyroidism. Was accepted as proper use, according to comedications as well: NSAID / inflammatory joint disease, corticosteroid / inflammatory or autoimmune disease, antibiotics / peptic ulcer disease by *H. pylori*, antiemetics / reflux or dispepsia. All others were considered off label comedications ^7^ ^,^ ^17^.

The daily defined dose (DDD) was used to measure the level of drug dispensation according to World Health Organization (WHO) recommendations, expressed as DHD (defined daily doses per 1000 inhabitants per day). The overall individual costs and the cost per 1000 inhabitants / day (CHD = (cost/365 x No. inhabitants) x (1000) for each PPI were used to estimate the economic impact of PPI use, using either the minimum or the maximum market price of each drug and the benchmark price of the dispensing company for different insurance companies (EPS); additionally, the monthly and annual costs of these prescriptions were estimated.

The protocol was reviewed by the Bioethics Committee of the Universidad Tecnológica of Pereira and the EPS participants, and was approved in the category of research without risk. For data analysis, we used the statistical package SPSS (r) Statistics Version 19 (IBM, USA) for Windows. The descriptive statistics used were mean, standard deviation, minimum and maximum values for continuous variables and percentages for categorical variables. We used the Chi-square test for the comparison of categorical variables. Binary logistic regression models were applied using nonjustified IPBs as the dependent variable. Statistical significance was predetermined to be *p*< 0.05 (95 % confidence interval).

## Results

At least one PPI was dispensed to 113,560 of the total 4.2 million users in the database on a monthly basis; of these patients, 65,460 (57.6%) were women, the mean age was 54.4 ± 18.7 years (range 1 to 100) and no significant differences in age between men and women (men's mean of 54.5/ vs. women's mean of 54.3). However, women were at greater risk of receiving a PPI for a non-indicated reason than men were (OR 1.4, 95% CI: 1.406 to 1.513, *p* = 0.03). The prevalence of PPI use was 3.0% in the studied population.

Table 1 summarizes the prescribing patterns of the main PPIs used in Colombia. The most prescribed PPI in the population was omeprazole (20 mg), followed by esomeprazole (20 mg and 40 mg), but other PPIs were also prescribed in lesser quantities. Of all the patients in the study, 107,952 were given a prescription for a single PPI (95.1%), while the remaining 5,608 (4.9%) were given prescriptions for a combination therapy with another antiulcer drug.

**Table 1:**
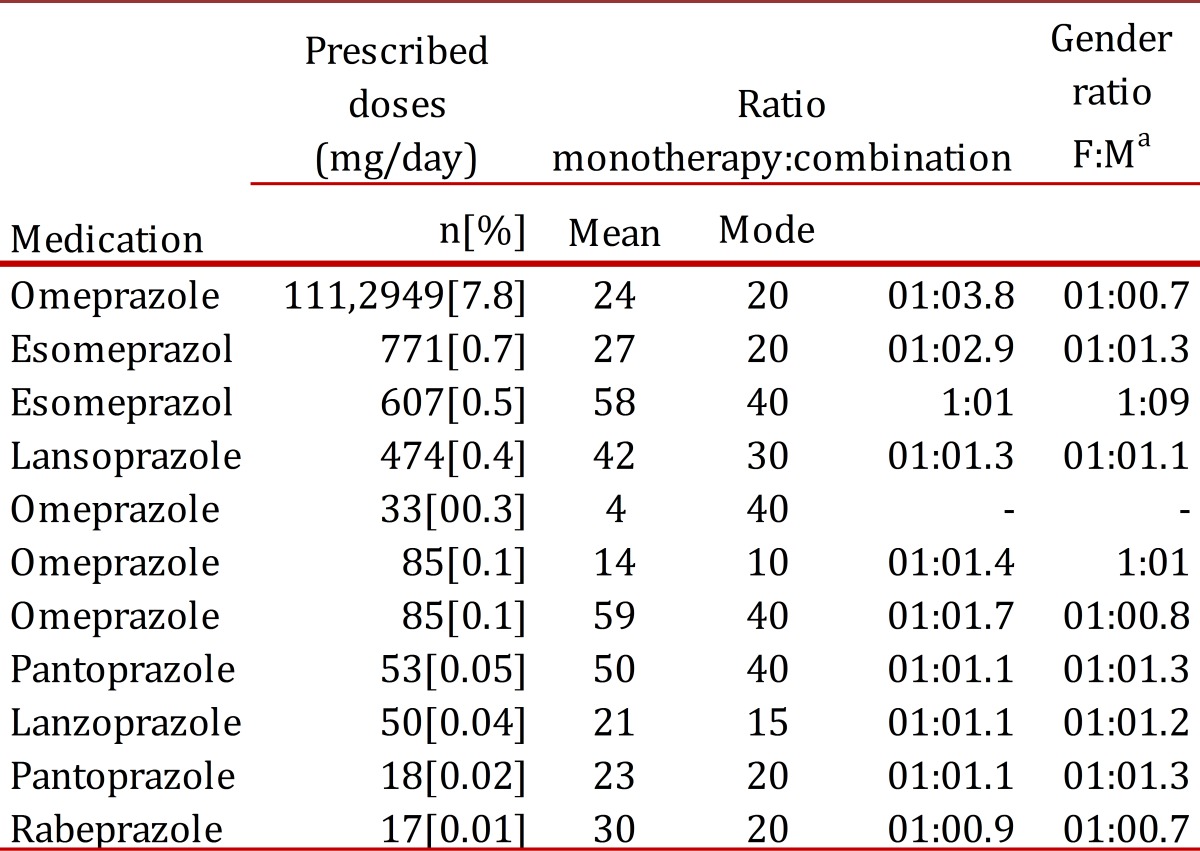
Proton Pump Inhibitors prescribing patterns in patients affiliated to the Colombian Health Social Security System (SGSSS), October 2010, a Female :Male, b Injectable .

### Comedication

Among the patients included in this study, 89,468 (78.8%) were concomitantly receiving one or more of the following groups of drugs that reflected a comorbidity and may cause drug interactions with PPIs: antihypertensives (53,552; 47.2% of patients), antithrombotics (28,534; 25.1%), analgesics (26,548; 23.4%), antidiabetic agents (13,019; 11.5%), NSAIDs (11,965; 10.5%), antiemetic agents (8,267; 7.3%), antibiotics (7,846; 6.9%); corticosteroids (6,645; 5.9%), lipid lowering drugs (6,135; 5.4%), DMARDs (3,749; 3.3%), nitrates (1,241; 1.1%), inotropic agents (785; 0.7%), bisphosphonates (652; 0.6%), thyroid-based medications (187; 0.2%), anti thyroid-based medications (108; 0.1%) and Cox-2 selective NSAIDs (37; <0.1%).

A total of 24,092 (21.2%) patients were receiving omeprazole exclusively, while 36,182 (31.9%) were receiving one additional drug besides omeprazol. A total of 29,733 (26.2%) received two additional drugs, 16,628 (14.6%) received three additional drugs, 5,488 (4.8 %) received four additional drugs, and 1,186 (1.0%) received five additional drugs. Other patients received as many as six to nine additional drugs (0.2%). The convenience of the use of PPIs along with additional drugs was reviewed. Their use was indicated in 87,349 (76.8%) of the cases reviewed, but was not medically justified in 26,211 (23.1%) of the cases.

In a binary logistic regression analysis, the use of analgesics, antidiabetics, antihypertensives, lipid lowering drugs and other anti-ulcer drugs were significantly associated with non-indicated PPI use. Alternatively, the use of antibiotics, NSAIDs, anti-emetics, corticosteroids and anti-platelet agents were significantly associated with appropriate PPI use (Table 2).

**Table 2.**
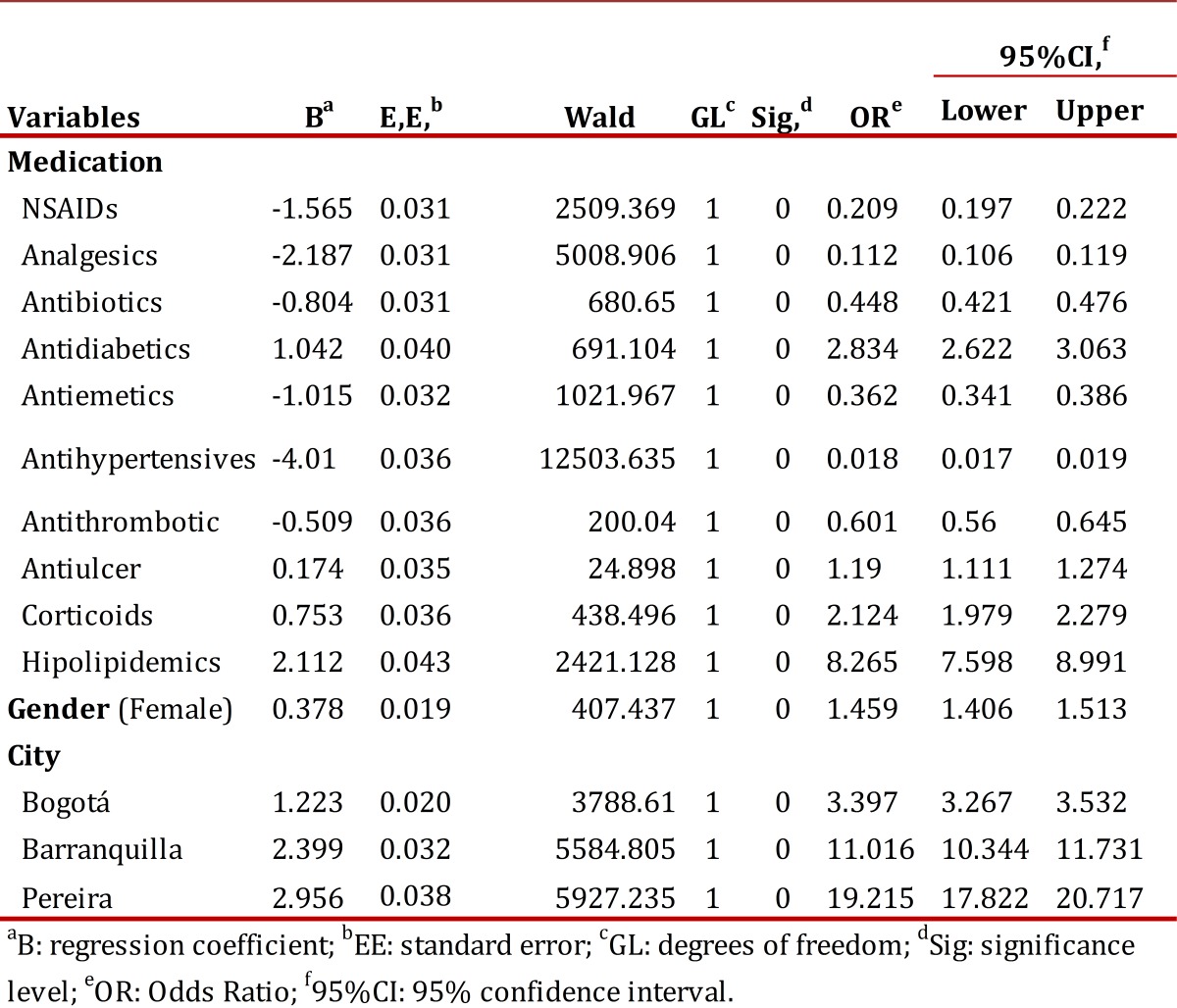
Variables related to justified and non-justified treatment with Proton Pump Inhibitors in binary logistic regression models, Colombia October, 2010. aB: regression coefficient; bEE: standard error; cGL: degrees of freedom; dSig: significance level; eOR: Odds Ratio; fCI 95%: 95% confidence interval.

### Description by cities

We also assessed the role of demographic variables in PPI prescribing practices among the 89 Colombian cities included in this analysis. Because some cities had only a few enrolled patients, Table 3 only includes the nine largest cities, which account for 81.0% of the patients. The same analysis was used for the global sum. As shown, no significant differences in the demographic variables, frequency of monotherapy use, comedication use and DDD were observed between the different cities. However, binary logistic regression analysis showed that in Bogota, Barranquilla and Pereira, there was a statistically significant association with the non-indicated use of PPIs (Table 2).

**Table 3:**
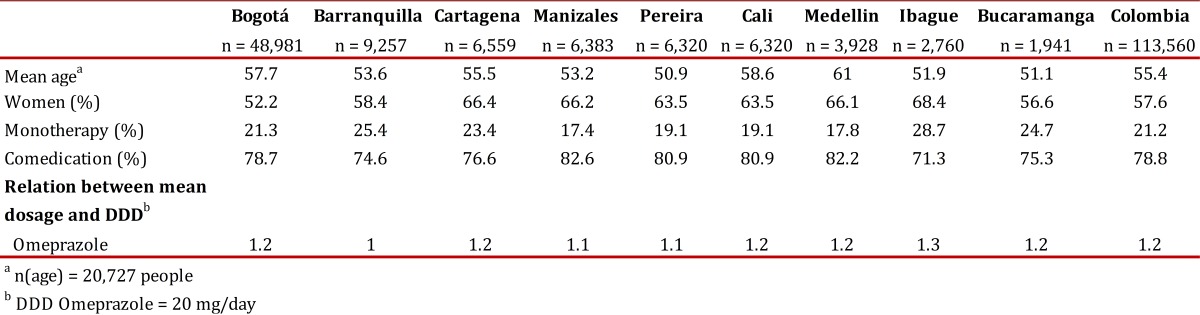
Comparison of socio-demographic variables and PPI prescription indicators among nine Colombian cities, October, 2010. a n(age) = 20,727 people b DDD Omeprazole = 20 mg/day

### Economic analysis

"It was found that, on average, the general population consumed 0.9 DDD of omeprazole, 0.01 DDD of esomeprazole and 0.004 DDD of lansoprazole per 1,000 inhabitants per day; this data is of clear possible use for future research comparisons. It was determined that the cost per 1000 inhabitants per day for omeprazole was U.S. $ 1.2, for esomeprazole U.S. $ 0.6, for lansoprazole U.S. $ 0.2, for pantoprazole U.S. $ 0.02, and for rabeprazole U.S. $ 0.01.

The estimated range in the monthly cost of all justified prescriptions was between U.S. $ 137,891, as calculated based on the cheapest omeprazole brand on the market (U.S. $ 0.04/pill), and U.S. $ 7,002,798, as calculated based on the most expensive omeprazole brand on the market (U.S. $ 2.9/pill).

The estimated monthly cost of non-justified prescriptions was U.S. $ 183,549 per month, of which 92.6% were for non-indicated uses of omeprazole and 7.4% for non-indicated uses of other PPIs. The annual justified cost was estimated to be U.S. $ 1,654,441 and the annual non-justified cost was estimated to be U.S. $ 2,130,131 based on minimum prices (Table 4). (Exchange rates were representative of the market in October of 2010, and were 1,816 pesos to 1 dollar).

**Table 4:**
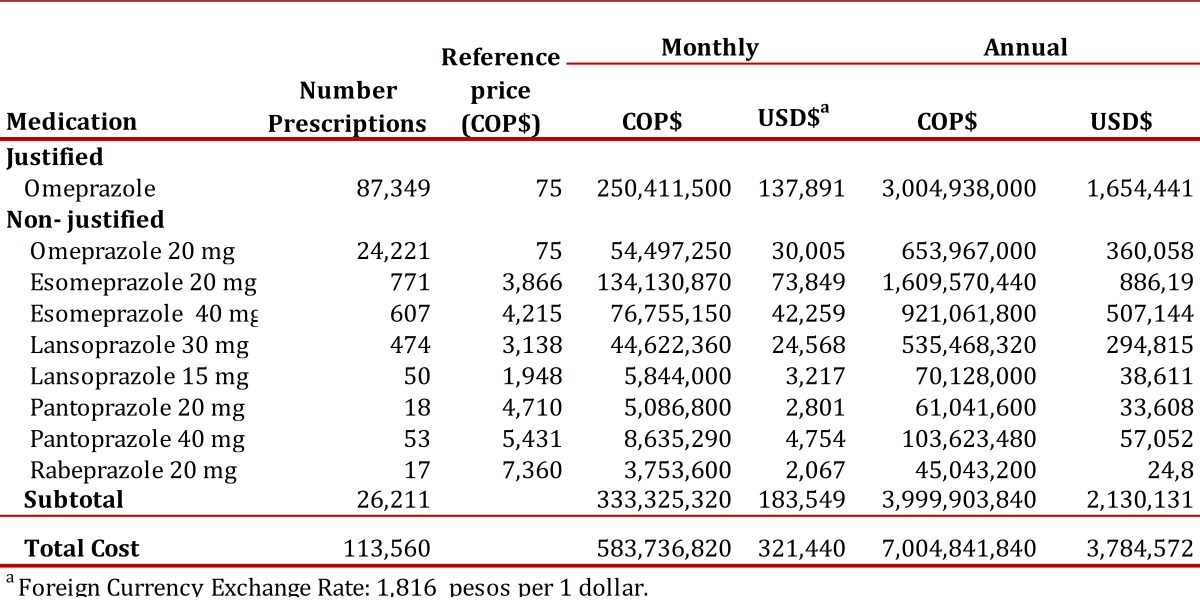
Monthly and annual cost of justified and non-justified PPI prescriptions in patients affiliated to the Colombian Health Social Security System (SGSSS), October 2010. a Foreign Currency Exchange Rate: 1,816 pesos per 1 dollar

## Discussion

Since PPIs are known to be more efficacious than other anti-ulcer medications and to have a relatively low toxicity, they have become one of the most prescribed drugs worldwide ^2^ ^,^ ^6^ ^,^ ^7^. 

The average age of patients enrolled in this study is greater than 54 years, with a female: male ratio of 1.36:1. Interestingly, female patients were found to be at higher risk of receiving an unjustified PPI. This may be because women tend to consult physicians more often and sooner than do men.

It is noteworthy that only omeprazole is included in the list of essential medicines in Colombia, and as such is considered the drug of choice for the treatment of acid peptic disease^18^. Given its placement on the essential medication list, it is understandable that 97.8% of patients were treated with omeprazole, while only a small group received another PPI. This result differs from other reports, which showed increased consumption of PPIs other than omeprazole^3^ ^,^ ^19^.

Prescribed doses of omeprazole and other PPIs are appropriate in the recommended dosage ranges for monotherapy. It is striking that about 5.0% of patients received anti-ulcer associations, given the limited evidence that their combined use provides any additional therapeutic benefits^20^. However, an evaluation of the use of PPIs indicated that 21.2% of patients received only an anti-ulcer medication, which is unusual given that 80% to 90% of all peptic ulcer diseases are associated with *H. pylori* infection. Such patients should therefore be receiving a PPI in conjunction with antibiotics, which was only documented in 6.9% of the cases analyzed^21^.

When assessing the comorbidities leading to the prescription of comedications, it was found that hypertension and diabetes are the most prevalent comorbidities, with 47.2% and 11.5% of individuals affected respectively. Such patients must therefore use drugs that are not associated with gastric or duodenal mucosal injury^14^. We also found that frequent use of concomitant antiplatelet medications with PPIs causes an increased risk of gastrointestinal bleeding^22^. An increased risk of bleeding is also associated with the use of NSAIDs and corticosteroids (10.5% and 5.9% of patients received comedication with a PPI, respectively)^22^ ^,^ ^23^. The remaining comedication patterns reported here, including comedication with lipid-lowering drugs and analgesics, have not been justified for use as mucosal protectors, especially given the highly aggressive nature of these drugs^7^.

However, it was found that PPIs were used appropriately in 76.9% of cases, particularly when prescribed along with NSAIDs, antiplatelet drugs, corticosteroids, antibiotics to eradicate *H. pylori* and prokinetics. This percentage is higher than that reported in previous studies demonstrating the inappropriate use of these drugs in patients with polymedication. The percentage of inappropriate PPI use was found to be 72.2% in Spain^6^, 66.1% in Argentina^8^, 13.0% in France^19^ and between 25 and 70% in the UK^5^.

It should be noted that the chronic use of PPIs is associated with an increased risk of both atrophic gastritis and community-acquired pneumonia^24^.

The differences in prescribing patterns between the different Colombian cities analyzed in this study, which included differences in the frequency of use, the percentage of monotherapy or comedication, and the DDD (Tables 2 and 3), are not surprising given the high variability in healthcare. Indeed, differences in prescribing habits are frequently reported in pharmacoepidemiological studies^15^ ^,^ ^16^. However, physicians in all cities typically prescribed an appropriate DDD. The most striking finding of this study is that patients in Pereira, Barranquilla and Bogota were 19.2, 11.0 and 3.3 times more likely to receive a prescription for a non-justified PPI use compared to patients from other cities. These variations, important for their clinical, social and economic implications, reflect the professional practice styles of physicians, which result from both personal factors and differences in medical training ^15^ ^,^ ^16^.

A 2002, an Irish study showed that 10.5% of drug sales covered under their health system, equivalent to about â‚55 million per year, were for the use of the commercial generic brand omeprazole ^25^. The findings of the present study are similar, given that when extrapolating the value of U.S. $ 3.7 million spent by 8.2% of the total Colombian population, the annual cost rises to about U.S. $ 46 million.

Furthermore, 83.6% of the annual non-justified costs are associated with the unjustified use of PPIs not included in the Basic Health Plan, meaning they are provided by commercial brands with higher retail prices. Simply replacing these brands with the generic omeprazole could result in annual savings of approximately U.S. $ 1.8 million, which would translate to population-level annual savings of about U.S. $ 20.7 million. An additional excess spending of U.S. $ 360,000 is caused by the non-justified use of generic omeprazole. In the total population, this spending reaches an annual cost about U.S. $ 4.4 million. To combat such problems, some countries have made proposals detailing the enormous savings that the replacement of commercial brands for generic brands would generate^19^.

There are some limitations to the present study and the interpretation of certain results therein. All data used for the analysis were obtained from dispensation databases rather than directly from either patients or prescribers or from a directly-consulted clinical record. This limitation will be mitigated by the second phase of this research, which will allow further characterization of the prescription of anti-ulcer drugs and particularly PPIs. Since this study design allows only for the collection of dispensation data, it will be necessary to gather additional information in further studies, including the indication for PPI use, the range of doses used, the incidence of adverse reactions attributable to medication, adherence to the recommended treatment, the degree of control of acid peptic disease and the associated morbidity levels. It should also be noted that the participants in this study represent a captive population given a list of specific available drugs, and therefore, the findings of this study are only applicable to populations with similar characteristics.

Based on the prescribing patterns recorded in this study, it can be stated that prescriptions for essential PPIs, mainly for their use in anti-ulcer monotherapy, were generally given at appropriate doses. Although a small number of patients received a PPI not on the list of essential medications, the annual cost of these in cases of non-justified use exceeds the entire annual cost of appropriately indicated omeprazole. Hypertension, cardiovascular risk prevention, pain management and diabetes were the most common conditions associated with taking a PPI. Dealing with the prescription of PPIs for indications outside of current recommendations will require interventions to improve the prescribing criteria for patients in the Colombian SGSSS.

Furthermore, it will be necessary to provide continuing education to physicians in order to ensure that they are up to date in their management of ulcers, especially with the use of PPIs. Such physicians must be trained to prescribe the appropriate medications in the appropriate doses, and educated about the conditions and comorbidities for which PPI use is indicated or contraindicated^7^ ^-^ ^17^. 

Finally, a similar study should address the prescribing practices of PPIs to hospitalized patients, as they represent a unique group associated with their own specific problems.
